# Hall Effect at the Focus of an Optical Vortex with Linear Polarization

**DOI:** 10.3390/mi14040788

**Published:** 2023-03-31

**Authors:** Victor V. Kotlyar, Alexey A. Kovalev, Elena S. Kozlova, Alexey M. Telegin

**Affiliations:** 1 Laser Measurements Laboratory, Image Processing Systems Institute of the RAS—Branch of FSRC “Crystallography & Photonics” of the RAS, 151 Molodogvardeyskaya St., 443001 Samara, Russia; kotlyar@ipsiras.ru (V.V.K.); alanko.ipsi@mail.ru (A.A.K.); 2 Technical Cybernetics Department, Samara National Research University, 34 Moskovskoe Shosse, 443086 Samara, Russia; talex85@mail.ru

**Keywords:** optical vortex, angular momentum, spin angular momentum, orbital angular momentum, topological charge, Hall effect, Richards–Wolf formulas, Poynting vector, energy flow, tight focusing

## Abstract

The tight focusing of an optical vortex with an integer topological charge (TC) and linear polarization was considered. We showed that the longitudinal components of the spin angular momentum (SAM) (it was equal to zero) and orbital angular momentum (OAM) (it was equal to the product of the beam power and the TC) vectors averaged over the beam cross-section were separately preserved during the beam propagation. This conservation led to the spin and orbital Hall effects. The spin Hall effect was expressed in the fact that the areas with different signs of the SAM longitudinal component were separated from each other. The orbital Hall effect was marked by the separation of the regions with different rotation directions of the transverse energy flow (clockwise and counterclockwise). There were only four such local regions near the optical axis for any TC. We showed that the total energy flux crossing the focus plane was less than the total beam power since part of the power propagated along the focus surface, while the other part crossed the focus plane in the opposite direction. We also showed that the longitudinal component of the angular momentum (AM) vector was not equal to the sum of the SAM and the OAM. Moreover, there was no summand SAM in the expression for the density of the AM. These quantities were independent of each other. The distributions of the AM and the SAM longitudinal components characterized the orbital and spin Hall effects at the focus, respectively.

## 1. Introduction

In 1909, Poynting [1] predicted that left-handed circularly polarized light has a spin angular momentum (SAM), or in short, a spin of −1, and right-handed circularly polarized light has a spin of +1. More precisely, he predicted that each photon could have a spin equal to Planck’s constant: either −ℏ or ℏ. In 1936, Beth [2] verified this experimentally by showing that when linearly polarized light passes through a quarter-wave plate, the plate acquires a torque. In 1992, Allen showed [3] that light, including each photon with a vortex phase described by the angular harmonic exp(*inφ*), has an orbital angular momentum (OAM) ℏn, where *n* is the topological charge (TC). In the paraxial case, the SAM and OAM are independent and are preserved separately during light propagation in free space. However, spin-orbit conversion (SOC) can occur when light is sharply focused near the focus [4]. So far, a lot of studies have been devoted to the investigation of SAM, OAM, and SOC [5]. Paper [5] is a small review of SOC in a tight focus of structured light. In [6], the tight focusing of radially polarized light was studied. In this work, it was shown that the intensity of the longitudinal light component at the focus increases with increasing numerical aperture, and becomes equal to the intensity of the transverse component at a unit numerical aperture. The Hall effect was investigated at the focus of an optical vortex with radial polarization [7]. It was shown in [7] that for the tight focusing of an optical vortex with radial polarization, the SAM is positive at the focus near the optical axis if the TC of the vortex is +1, and the SAM is negative if the TC of the vortex is −1. This is the so-called catalyst-like effect. In [8], the authors showed that when focusing an optical vortex with radial polarization, the longitudinal projection of the SAM vector has different signs at different distances from the optical axis in the focal plane. This is the radial spin Hall effect. In [9], the 3D SAM was studied in the tight focus of an optical vortex with linear polarization. In this study, the force vector was calculated that will act on an ellipsoidal particle that is in focus. The tight focusing of an optical vortex with azimuthal polarization was observed in [10]. It was shown in [10] that when the optical vortex TC sign changes near the optical axis in the focus, the sign of the SAM longitudinal projection also changes, and therefore, the particle placed in the focus changes the rotation direction around its own axis and around the optical axis. In [11], the angular momentum (AM) in a sharp focus of hybrid cylindrical vector beams was studied. It was shown in [11] that for such light fields, the longitudinal component of the SAM is equal to zero at the focus. The orbital motion of microparticles in a tight focus of optical vortices with circular and radial polarization was investigated in [12]. In [13], SOC was considered in nonparaxial beams with hybrid polarization. It was shown in [13] that when light with a high-order hybrid polarization is tightly focused, regions are formed in the focus in which the longitudinal components of the OAM and the SAM change signs. That is, the spin and orbital Hall effects take place. Beams with hybrid polarization in a tight focus were observed in [14]. It was shown in [14] that when focusing light whose polarization changes only along the radius, the polarization in the focal plane will also change along the radius. Linear polarization and elliptical polarization will alternate but have the same sign. In [15], the paraxial focusing of Bessel beams with circular polarization was studied. It was shown in [15] that the sign of the angular momentum vector will be different on different sides of the light intensity ring in the beam. The tight focusing of high-order Poincare beams was considered in [16]. In our recent works, we investigated the Hall effect in the tight focus of high-order cylindrical vector beams [17], beams with hybrid inhomogeneous polarization [18], Poincaré beams [19], and optical vortices with circular polarization [20]. Another version of the Hall effect in a sharp focus appears when the center of a vortex laser beam gravity is shifted in the case of its limitation by a diaphragm [21]. The Hall effect in the tight focus of an optical vortex with linear polarization has not been considered before.

We note that the spin Hall effect arises not only in a tight focus but also when light is scattered by inhomogeneous structures. Thus, it was shown theoretically in [22] and experimentally in [23] that when a laser beam with linear polarization is reflected from a microresonator with Bragg mirrors, four regions with circular polarization of different signs are formed in the beam. Furthermore, in [24], it was experimentally shown that the scattering of a Hermite–Gaussian (HG_0,1_) beam with linear polarization on a silver nanowire (AgNW) also gives the spin Hall effect. It was shown in [25] that due to SOC, gold particles placed in the tight focus of the Laguerre–Gaussian vortex beam (LG_0,1_) rotate at different speeds for light with left and right circular polarization.

In this study, we considered the tight focusing of an optical vortex with an integer TC and linear polarization. Using Richards–Wolf theory [26], which accurately describes light in the vicinity of a tight focus of coherent light, exact analytical expressions were obtained for the longitudinal components of the SAM, OAM, and AM vectors in the focal plane for an optical vortex with linear polarization. It was shown that the longitudinal SAM and OAM components averaged over the beam cross-section were preserved in the initial and the focal planes. It was also demonstrated that there was a separation of regions with different signs of the SAM longitudinal component and regions with different signs of the AM longitudinal component at the focus. It was found that the AM and the SAM values were independent and sufficient to describe the light at the focus, while the meaning of the OAM value at the focus was not clear since the AM was not the sum of the SAM and OAM. However, it was easy to prove the conservation of the OAM value, while it was not possible to prove the conservation of the AM.

## 2. Components of the Electric and the Magnetic Fields and the Energy Flux at the Focus

Consider the initial Jones vector for an optical vortex with linear polarization:

(1) $$E_{n}\left(\varphi \right)=\exp\left(in\varphi \right)\left(\begin{array}{l} 1\\ 0 \end{array}\right),$$

where (*r*, *φ*) are polar coordinates in the beam cross-section, *n* is the TC and is an integer, and the linear polarization vector is directed along the horizontal *x*-axis. In [27], the electric and magnetic field components in the plane of tight focus for the initial field (1) were obtained:

(2) $$\begin{array}{l} E_{x}=\frac{i^{n-1}}{2}e^{in\varphi }\left(2I_{0,n}+e^{2i\varphi }I_{2,n+2}+e^{-2i\varphi }I_{2,n-2}\right),\\ E_{y}=\frac{i^{n}}{2}e^{in\varphi }\left(e^{-2i\varphi }I_{2,n-2}-e^{2i\varphi }I_{2,n+2}\right),\\ E_{z}=i^{n}e^{in\varphi }\left(e^{-i\varphi }I_{1,n-1}-e^{i\varphi }I_{1,n+1}\right),\\ H_{x}=\frac{i^{n}}{2}e^{in\varphi }\left(e^{-2i\varphi }I_{2,n-2}-e^{2i\varphi }I_{2,n+2}\right),\\ H_{y}=\frac{i^{n-1}}{2}e^{in\varphi }\left(2I_{0,n}-e^{2i\varphi }I_{2,n+2}-e^{-2i\varphi }I_{2,n-2}\right),\\ H_{z}=i^{n+1}e^{in\varphi }\left(e^{-i\varphi }I_{1,n-1}+e^{i\varphi }I_{1,n+1}\right). \end{array}$$

Formula (2) includes functions *I*_*ν*,*μ*_ that depend only on the radial variable *r*:

(3) $$\begin{array}{l} I_{\nu ,\mu }=2kf\int_{0}^{\alpha }\sin^{\nu +1}\left(\frac{\theta }{2}\right)\cos^{3-\nu }\left(\frac{\theta }{2}\right)\cos^{1/2}\left(\theta \right),\\ \;\;\;\;\times A\left(\theta \right)e^{ikz\cos\theta }J_{\mu }\left(kr\sin\theta \right)d\theta , \end{array}$$

where *k* = 2π/λ is the wavenumber of monochromatic light with a wavelength of λ; *f* is the focal length of the lens; α is the maximum angle of the rays’ inclination to the optical axis, which determines the numerical aperture (NA) of the aplanatic lens NA = sin α; and *J_μ_*(*kr*sin*θ*) is the *μ*th order Bessel function of the first kind. In Equation (2) and everywhere below, the indices *ν* and *μ* can take the following values: *ν =* 0, 1, 2; *μ* = *n* − 2, *n* − 1, *n*, *n* + 1, *n* + 2. *A*(*θ*) is a real function that determines the radially symmetric initial field amplitude, which depends on the inclination angle *θ* of the beam emanating from a point on the initial spherical front and converging to the center of the focus plane. The description of the light field at the focus using (3) was obtained for the first time in the classic study by Richards and Wolf [26]. Next, we found the components of the Poynting vector:

(4) P=c2πReE*×H

where **E** and **H** are vectors of the electric and the magnetic fields, the signs “*” and “×” mean complex conjugation and vector product, Re is the real part of a complex number, and *c* is the speed of light in a vacuum. In the following, we omitted the constant *c*/(2π). Substituting (2) into (4), we obtained the following at the focus of the field in polar coordinates (1):

(5) $$\begin{array}{l} P_{r}=0,\\ P_{\varphi }=Q\left(r\right),\\ P_{z}=\frac{1}{2}\left(2I_{0,n}^{2}-I_{2,n+2}^{2}-I_{2,n-2}^{2}\right),\\ Q\left(r\right)=I_{1,n+1}\left(I_{0,n}+I_{2,n+2}\right)+I_{1,n-1}\left(I_{0,n}+I_{2,n-2}\right). \end{array}$$

It follows from (5) that the transverse energy flux at the focus of the field (1) rotates counterclockwise if *Q*(*r*) > 0 and clockwise if *Q*(*r*) < 0. The longitudinal component of the energy flow at different radii *r* can be positive or negative. It can be shown that the total energy of each term in *P_z_* at the focus is equal to the expression:

(6) $$\begin{array}{l} W_{\nu ,\mu }=2\pi \int_{0}^{\infty }{\left|I_{v,\mu }\left(r\right)\right|}^{2}rdr\\ =4\pi f^{2}\int_{0}^{\alpha }\sin^{2\nu +1}\left(\frac{\theta }{2}\right)\cos^{5-2v}\left(\frac{\theta }{2}\right){\left|A\left(\theta \right)\right|}^{2}d\theta =W_{\nu } \end{array}$$

Equation (6) was obtained using Equation (3) and the orthogonality of the Bessel functions:

$$\int_{0}^{\infty }J_{\mu }\left(k\sin\theta r\right)J_{\mu }\left(k\sin\theta \prime r\right)rdr==\frac{1}{k^{2}}\frac{\delta \left(\sin\theta -\sin\theta \prime \right)}{\sin\theta }.$$

It can be seen from (6) that the energy (or power) does not depend on the order of the Bessel function *μ*. Applying Formula (6) to the axial energy flow crossing the focus (5), we obtain

(7) $$\begin{array}{l} {\hat{P}}_{z}=\int_{0}^{\infty }rdr\int_{0}^{2\pi }d\varphi P_{z}\left(r\right)=W_{0}-W_{2}\\ =W-2W_{1}-2W_{2}. \end{array}$$

In (6), *W* is the total power of the laser beam. It can be shown that the power *W*_0_ is approximately seven times greater than the power *W*_2_ (it is exactly seven times greater for α = π/2 and for |*A*(θ)| ≡ 1). Therefore, the total flow (7) is always positive, although the energy flux density (5) at different radii *r* can be both positive and negative (reverse energy flux [28]). Equation (7) shows that not all of the power *W* crosses the focus plane from left to right (in the positive direction of the *z*-axis). The part of the power 2*W*_1_ propagates in the direction perpendicular to the optical axis and does not cross the focus plane. Part of the power *W*_2_ crosses the focus plane in the opposite direction and another part of the power *W*_0_ flows along the positive direction of the *z*-axis. It is interesting that the power ratio (7) does not depend on the TC of the beam (1).

## 3. The Longitudinal Component of the SAM Vector at the Focus

Next, we found the axial SAM component, which shows the presence of light with elliptical and circular polarization in the focus. The longitudinal SAM component is defined as follows [29]:

(8) Sz=2ImEx*Ey,

where Im is the imaginary part of a complex number. Substituting (2) into (8), we obtained the axial SAM component at the focus for the field (1):

(9) $$S_{z}=\frac{1}{2}\left(I_{2,n+2}-I_{2,n-2}\right)\left(I_{2,n+2}+I_{2,n-2}+2\cos(2\varphi )I_{0,n}\right).$$

It can be seen from (9) that if the first factor is not equal to zero, then there are four regions in the focus plane where the SAM sign is different. The centers of these areas lie on the Cartesian axes: two regions centered on the vertical axis and two regions centered on the horizontal axis. If $I_{2,n+2}-I_{2,n-2}$ > 0, then at *φ* = 0 and *φ* = π, the second factor is positive and *S_z_* > 0, while at *φ* = π/2 and *φ* = 3π/4, the second factor in (9) is negative and *S_z_* < 0. If, conversely, $I_{2,n+2}-I_{2,n-2}$ < 0, then the SAM is positive on the vertical axis and negative on the horizontal axis. The first factor is equal to zero only in the absence of an optical vortex (*n* = 0). Thus, it follows from (9) that the spin Hall effect takes place for the field (1) at the focus at *n* ≠ 0. It leads to the separation of the vectors with left and right elliptical polarizations (with different spins) from each other and their localization in four regions in pairs on the vertical and horizontal axes. Since the axial SAM component in the initial plane (1) is equal to zero (due to linear polarization), then the total spin at the focus must be equal to zero. Indeed, when we integrated the SAM in (9), we obtained

(10) $$\begin{array}{l} {\hat{S}}_{z}=\int_{0}^{\infty }rdr\int_{0}^{2\pi }d\varphi S_{z}\left(r,\varphi \right)\\ =\frac{1}{2}\int_{0}^{\infty }rdr\int_{0}^{2\pi }d\varphi \left(I_{2,n+2}^{2}-I_{2,n+2}^{2}-2\cos2\varphi I_{0,n}(I_{2,n-2}-I_{2,n+2})\right).\\ =\frac{1}{2}\left(W_{2}-W_{2}+0\right)=0. \end{array}$$

Integration over the entire focus plane of the first and second terms gives the difference between two identical energies (6). The third term depending on cos(2*φ*) is zero when integrated over an integer number of the angle *φ* periods. Since the total spin at the focus is zero, regions with different spins must appear in pairs to cancel each other out. 

## 4. The Intensity and the Longitudinal OAM Component at the Focus

Next, we found the longitudinal component of the OAM vector at the focus of the field (1). The longitudinal OAM component is equal to the following expression [20]:

(11) Lz=ImEx*∂∂φEx+Ey*∂∂φEy+Ez*∂∂φEz,

Substituting (2) into (11), we obtained

(12) $$\begin{array}{l} L_{z}=\frac{1}{2}\left\{2nI_{0,n}^{2}+2\left(n+1\right)I_{1,n+1}^{2}+2\left(n-1\right)I_{1,n-1}^{2}\right.\\ +\left(n+2\right)I_{2,n+2}^{2}+\left(n-2\right)I_{2,n-2}^{2}+2\cos\left(2\varphi \right)\\ \left.\times \left[2\left(n+1\right)I_{0,n}I_{2,n+2}+2\left(n-1\right)I_{0,n}I_{2,n-2}-nI_{1,n-1}I_{1,n+1}\right]\right\}. \end{array}$$

Since expression (12) has the form $L_{z}=A\left(r\right)+\cos\left(2\varphi \right)B\left(r\right)$, then it has two maxima on the horizontal axis at *φ* = 0 and *φ* = π and two minima on the vertical axis at *φ* = π/2 and *φ* = 3π/4. It can be shown that the main contribution is made by terms containing the integral in (3) with a zero first index, that is, expression (12) can be written approximately as follows:

(13) $$\begin{array}{l} L_{z}\approx nI_{0,n}^{2}+2\cos\left(2\varphi \right)I_{0,n}\left(\left(n+1\right)I_{2,n+2}+\left(n-1\right)I_{2,n-2}\right)\approx \\ nI_{0,n}\left(I_{0,n}+4I_{2,n+2}\cos\left(2\varphi \right)\right). \end{array}$$

It can be seen from (13) that at *φ* = 0 and *φ* = π, there are regions with *L_z_* > 0 in focus, and at *φ* = π/2 and *φ* = 3π/2, there are regions with *L_z_* < 0. That is, there is a spatial separation of the OAM with different signs at the focus of the field (1). Moreover, the location in the focal plane of these four regions with centers on the horizontal and vertical axes correlates with areas of elliptical polarization with different signs (9). It should be noted that in the initial plane (1), the OAM axial component (11) is equal to *L_z_ = nW,* where *W* is the total beam power. When we integrated (12) over the entire focal plane, we found that the terms containing cos(2*φ*) disappeared since the integration over the angle was performed over an integer number of periods. Integration of other terms led to the following expression:

(14) $$\begin{array}{l} {\hat{L}}_{z}=\int_{0}^{\infty }rdr\int_{0}^{2\pi }d\varphi L_{z}\left(r,\varphi \right)\\ =\frac{1}{2}\int_{0}^{\infty }rdr\int_{0}^{2\pi }d\varphi (2nI_{0,n}^{2}+2\left(n+1\right)I_{1,n+1}^{2}+2\left(n-1\right)I_{1,n-1}^{2}+\left(n+2\right)I_{2,n+2}^{2}+\left(n-2\right)I_{2,n-2}^{2})\\ =nW_{0}+\left(n+1\right)W_{1}+\left(n-1\right)W_{1}+\frac{1}{2}\left(n+2\right)W_{2}+\frac{1}{2}\left(n-2\right)W_{2}=n\left(W_{0}+2W_{1}+W_{2}\right)=nW. \end{array}$$

The last equality in (14) follows from the power balance of the entire beam and its components at the focus. The balance can be obtained by integrating the intensity distribution over the entire beam cross-section. The intensity distribution at the focus follows from (4) and is equal to

(15) $$\begin{array}{l} I=\frac{1}{2}\left[2I_{0,n}^{2}+I_{2,n+2}^{2}+I_{2,n-2}^{2}+2I_{1,n+1}^{2}+2I_{1,n-1}^{2}\right.\\ +\left.2\cos\left(2\varphi \right)\left(I_{0,n}I_{2,n+2}+I_{0,n}I_{2,n-2}-2I_{1,n+1}I_{1,n-1}\right)\right]. \end{array}$$

We integrated expression (15) for the intensity over the entire beam cross-section at the focus and obtained

(16) $$\begin{array}{l} W=\int_{0}^{\infty }\int_{0}^{2\pi }I\left(r,\varphi \right)rdrd\varphi =\frac{1}{2}\int_{0}^{\infty }\int_{0}^{2\pi }rdrd\varphi \left[2I_{0,n}^{2}+I_{2,n+2}^{2}+I_{2,n-2}^{2}+\right.\\ 2I_{1,n+1}^{2}+2I_{1,n-1}^{2}+\left.2\cos\left(2\varphi \right)\left(I_{0,n}I_{2,n+2}+I_{0,n}I_{2,n-2}-2I_{1,n+1}I_{1,n-1}\right)\right]\\ =W_{0}+W_{2}+2W_{1}. \end{array}$$

To obtain (16), Equation (6) and the fact that the integration of the term with cos(2*φ*) over the period gives zero were used. It can be seen from (16) that the total beam power is equal to

(17) $$W=W_{0}+W_{2}+2W_{1}.$$

Equation (17) was used in the last step of (14). Thus, we showed that the longitudinal OAM component averaged over the beam cross-section was preserved for the field in (1). Preservation of the full OAM during propagation of the beam (1) was the reason for the formation of an even number of regions in the focus, in which the OAM component had a different sign (the orbital Hall effect).

## 5. The Longitudinal Component of the AM Vector at the Focus

Next, we compared the longitudinal components of the AM and of the sum of SAM and OAM. The AM is given by the equation [30]:

(18) J=r×P.

The longitudinal AM component is determined only by the angular component of the energy flux at the focus (5) and is equal to

(19) $$J_{z}=rQ\left(r\right)=r[I_{1,n+1}\left(I_{0,n}+I_{2,n+2}\right)+I_{1,n-1}\left(I_{0,n}+I_{2,n-2}\right)].$$

From (19), it can be seen that the AM longitudinal component on the optical axis is always equal to zero since the “leverage” is equal to zero. We compared expression (19) with the sum of SAM (9) and OAM (12) for the field (1) in the focus:

(20) $$\begin{array}{l} S_{z}+L_{z}=\frac{1}{2}\{2nI_{0,n}^{2}+2\left(n+1\right)I_{1,n+1}^{2}+2\left(n-1\right)I_{1,n-1}^{2}\\ +\left(n+1\right)I_{2,n+2}^{2}+\left(n-1\right)I_{2,n-2}^{2}+2\cos\left(2\varphi \right)\\ \times [\left(4n+3\right)I_{0,n}I_{2,n+2}+\left(4n-3\right)I_{0,n}I_{2,n-2}-2nI_{1,n+1}I_{1,n-1}]\}. \end{array}$$

A comparison of (19) and (20) shows that the AM is not equal to the sum of the SAM and OAM. For example, the angular momentum (19) is radially symmetric and does not depend on the angle *φ*, while the sum of SAM and OAM (20) depends on the azimuth angle as cos(2*φ*). Therefore, there must be a third term *X_z_*, which must be added to the sum (20) in order for the equality to hold:

(21) $$J_{z}=S_{z}+L_{z}+X_{z}$$

Several questions arise from this information. What is transferred to the particle and causes it to rotate along a circular path: AM (19) or OAM (12)? Furthermore, what should be called the orbital Hall effect: separation of regions with different OAM signs (12) or AM signs (19)? Most likely, the orbital Hall effect is determined by the different directions of the transverse energy flow (5) since the transverse flow “catches” the microparticle and forces it to rotate along the “orbit” [31]. Therefore, the AM, which is proportional to the transverse energy flux *Q*(*r*), is responsible for the rotation of the particle along a circular trajectory.

## 6. Physical Meaning of the Third Term in the Equation for the AM

In this section, we show that the terms SAM and OAM in (21) were formed artificially and that only two characteristics are sufficient for the light field. These characteristics are SAM and AM, which are not related to each other. We start with the definition of the AM (18) and explicitly write out the quantities included in it:

(22) J=r×P=Im(r×(E∗×∇×E)

In (22), all dimensional constants are omitted. Further, for definiteness, we consider obtaining the longitudinal component of the SAM vector in Cartesian coordinates. From (22) we obtained

(23) Jz=ImxEx∗∂Ex∂y−∂Ey∂x+Ez∗∂Ez∂y−∂Ey∂z−yEy∗∂Ey∂x−∂Ex∂y+Ez∗∂Ez∂x−∂Ex∂z=ImxEx∗∂Ex∂y+Ez∗∂Ez∂y−Ex∗∂Ey∂x−Ez∗∂Ey∂z−yEx∗∂Ey∂x+Ez∗∂Ez∂x−Ey∗∂Ex∂y−Ez∗∂Ex∂z.

Let us write in a general form the expression for the OAM longitudinal component (11), but using Cartesian coordinates:

(24) Lz=ImEx∗x∂∂y−y∂∂xEx+Ey∗x∂∂y−y∂∂xEy+Ez∗x∂∂y−y∂∂xEz=ImxEx∗∂Ex∂y+Ey∗∂Ey∂y+Ez∗∂Ez∂y−yEx∗∂Ex∂x+Ey∗∂Ey∂x+Ez∗∂Ez∂x.

Comparing (23) and (24), there are four terms at *x* and *y* in (23) and three terms at *x* and *y* in (24). Therefore, in order to form a separate term in (23) as in (24), we added and subtracted two terms in (23). Then, instead of (23), we obtained

(25) Jz=ImxEx∗∂Ex∂y+Ez∗∂Ez∂y+Ey∗∂Ey∂y−Ey∗∂Ey∂y−Ex∗∂Ey∂x−Ez∗∂Ey∂z−yEx∗∂Ey∂x+Ez∗∂Ez∂x+Ex∗∂Ex∂x−Ex∗∂Ex∂x−Ey∗∂Ex∂y−Ez∗∂Ex∂z.

The added terms in (25) are marked with angular parentheses. They do not change the value of the expression (23). Now, in (25), we grouped the terms in order to explicitly separate the term equal to *L_z_* (24):

(26) Jz=Lz−ImxEx∗∂Ey∂x+Ey∗∂Ey∂y+Ez∗∂Ey∂z−yEx∗∂Ex∂x+Ey∗∂Ex∂y+Ez∗∂Ex∂z.

Next, we added and subtracted the SAM longitudinal component (8) in (26) and obtained

(27) Jz=Lz+Sz−ImxEx∗∂Ey∂x+Ey∗∂Ey∂y+Ez∗∂Ey∂z−yEx∗∂Ex∂x+Ey∗∂Ex∂y+Ez∗∂Ex∂z+〈Ex∗Ey−Ey∗Ex〉=Lz+Sz+Xz.

Thus, from (23), we obtained (21). In (27), the difference between the two terms in small triangular brackets is equal to the SAM with the opposite sign. That is, OAM and SAM in the expression for AM were artificially formed by adding and subtracting additional terms. As a result, the third term *X_z_* appeared, which has no meaning in the general case. However, in some cases, a certain meaning can be attributed to it. For instance, if *L_z_* + *S_z_* = 0, then the term *X_z_* is equal to the angular momentum of the light field (*X_z_* = *J_z_*). The conclusion from this subsection is presented below. The orbital Hall effect occurs at the focus when the regions with the AM longitudinal component of different signs are separated, that is, the regions appear with a different direction of the transverse energy flow rotation. The spin Hall effect occurs at the focus when the regions with the SAM longitudinal component of different signs are separated from each other, that is, the regions in which the polarization vector rotates in different directions are separated.

## 7. Simulation

**Figure 1:**
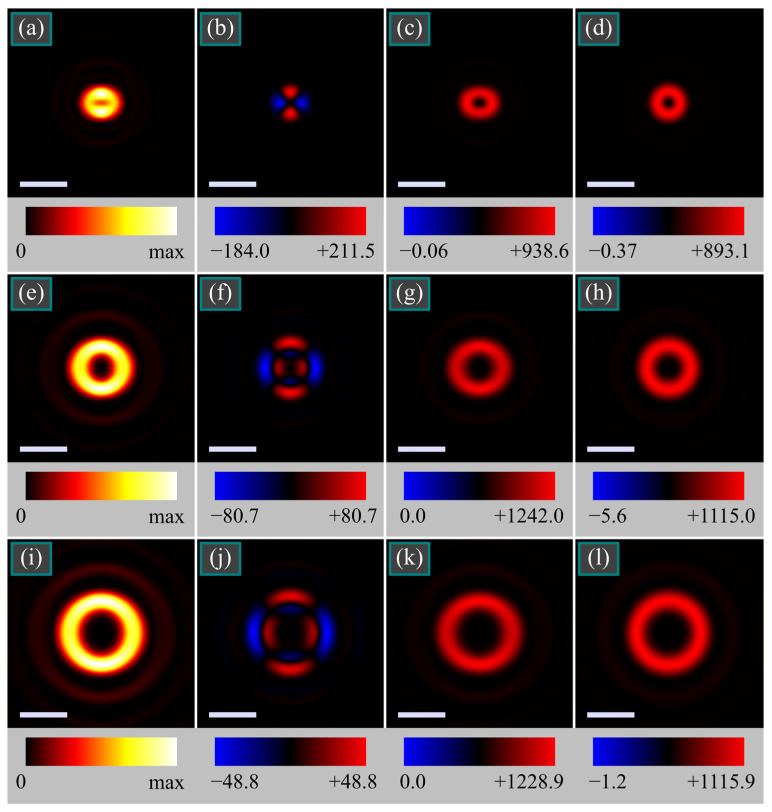
Distributions of the intensity (**a**,**e**,**i**), the SAM density (**b**,**f**,**j**), the OAM density (**c**,**g**,**k**), and the AM density (**d**,**h**,**l**) of the beam (1) in a tight focus at *n* = 1 (**a**–**d**), *n* = 3 (**e**–**h**), and *n* = 5 (**i**–**l**) with the following calculation parameters: wavelength λ = 532 nm, focal length *f* = 10 µm, numerical aperture NA = 0.95, and size of the computational domain = 4 × 4 μm^2^. The scale mark in all figures means 1 µm. The numbers on the color scales below each figure indicate the minimum and maximum values.

Figure 1 shows the distributions of the intensity, as well as the densities of the SAM, the OAM, and the AM of the beam (1) in a tight focus at *n* = 1 (Figure 1a–d), *n* = 3 (Figure 1e–h), and *n* = 5 (Figure 1i–l). Figure 1 confirmed Formula (9), according to which the maximum and minimum values of the SAM density were achieved on the Cartesian axes. Figure 1 also confirmed Formulas (9) and (13), according to which the OAM density was symmetric with respect to the Cartesian axes, and the AM density had radial symmetry.

It follows from Figure 1 that the spin Hall effect occurred at the focus (Figure 1b,f,j), when four local regions with positive and negative (approximately equal in absolute value) SAM were formed at different radii in the focal plane. The orbital Hall effect also took place at the focus (Figure 1d,h,l). However, first, it was radial, and, second, it was weakly expressed since the positive AM distributed over a ring of one radius was much larger by a modulus of a negative AM distributed over a ring of another radius. In Figure 1d,h,l, the blue ring with the negative AM was not visible, but the value of the negative AM is shown on the horizontal color scale.

**Figure 2:**
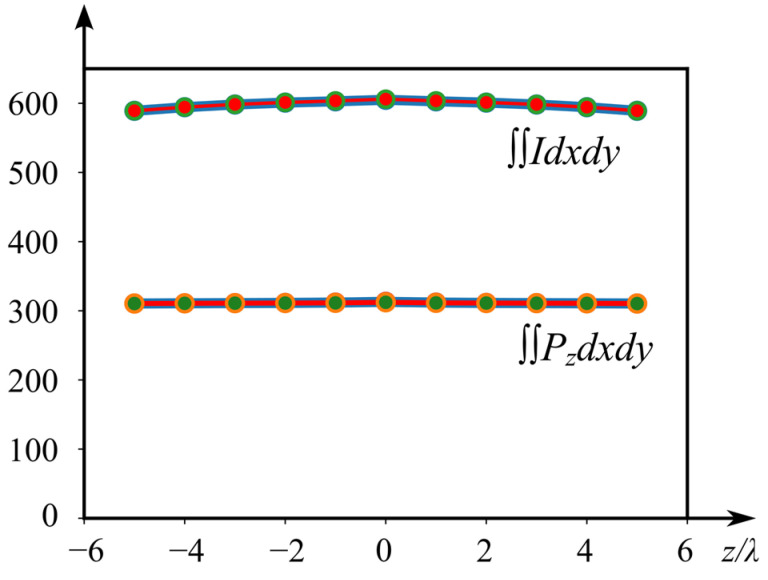
Dependence of the intensity and the longitudinal component of the Umov–Poynting vector, integrated over the transverse plane, on the distance to the focus *z*. The upper curve is the total intensity(power) and the lower curve is the total longitudinal power flow. The calculation parameters were the same as in Figure 1. The graphs show the curves for *n* = 1 and *n* = 3, but they almost coincide with each other. It was assumed that the distribution of the focused light was uniform (|*A*(θ)| ≡ 1). In this case, the total energy was 2π*f*^2^ ≈ 628 µm^2^. Numerically obtained values were approximately equal to ∫∫*Idxdy* ≈ 600 µm^2^. The theoretical value of the total longitudinal power flow was π*f*^2^ ≈ 314 µm^2^. Numerically obtained values were approximately equal to ∫∫*P_z_dxdy* ≈ 310 µm^2^.

Figure 2 illustrates the dependences of the total intensity (power) and the total longitudinal power flux on the distance to the focus.

Figure 2 confirmed Formula (17), according to which the total light energy (power) should be equal to 2π*f* ^2^ (at α = π/2 and |*A*(θ)| ≡ 1), and Formula (7), according to which the total longitudinal power flow should be equal to π*f* ^2^. That is, the representation of the longitudinal power flow through the forward flow, the perpendicular flow, and the reverse flow was also confirmed.

## 8. Discussion of Results

In this study, we showed that the longitudinal SAM and OAM components averaged over the focus plane for an initial optical vortex (with arbitrary radially symmetric real amplitude) with linear polarization are preserved separately. However, this is not true in all cases. For example, if we considered the tight focusing of an optical vortex with circular polarization [20], then the averaged axial SAM and OAM components were not conserved. Instead, only their sum was conserved. Indeed, the densities of the longitudinal components of the SAM and OAM vectors at the focus of an optical vortex with right-hand circular polarization

(28) $$E(\theta ,\varphi )=\frac{1}{\sqrt{2}}\exp(in\varphi )\left(\begin{array}{l} 1\\ i \end{array}\right)$$

have the form, respectively:

(29) $$S_{z}=I_{0,n}^{2}-I_{2,n+2}^{2},$$

(30) $$L_{z}=nI_{0,n}^{2}+\left(n+2\right)I_{2,n+2}^{2}+2\left(n+1\right)I_{1,n+1}^{2}.$$

We integrated both of these quantities (29) and (30) over the plane of the focus and obtained

(31) $$\begin{array}{l} {\hat{S}}_{z}=\int_{0}^{\infty }rdr\int_{0}^{2\pi }d\varphi S_{z}\left(r,\varphi \right)\\ =\int_{0}^{\infty }rdr\int_{0}^{2\pi }d\varphi \left(I_{0,n}^{2}-I_{2,n+2}^{2}\right)=\left(W_{0}-W_{2}\right), \end{array}$$

(32) $$\begin{array}{l} {\hat{L}}_{z}=\int_{0}^{\infty }rdr\int_{0}^{2\pi }d\varphi L_{z}\left(r,\varphi \right)\\ =\frac{1}{2}\int_{0}^{\infty }rdr\int_{0}^{2\pi }d\varphi \left(nI_{0,n}^{2}+\left(n+2\right)I_{2,n+2}^{2}+2\left(n+1\right)I_{1,n+1}^{2}\right)\\ =nW_{0}+\left(n+2\right)W_{2}+2\left(n+1\right)W_{1}=nW+2\left(W_{2}+W_{1}\right). \end{array}$$

Moreover, the sum of (31) and (32) is equal to

(33) $${\hat{S}}_{z}+{\hat{L}}_{z}=\left(n+1\right)W.$$

We obtained the following expressions for the SAM and the OAM in the initial plane:

(34) $${\hat{S}}_{z}=W,\;\;{\hat{L}}_{z}=nW,\;\;{\hat{S}}_{z}+{\hat{L}}_{z}=\left(n+1\right)W.$$

It can be seen from (33) that in the initial field for an individual photon, the sum of the spin and the OAM for the beam (28) was equal to $S_{z}+L_{z}=(n+1)\hbar$, while for the entire beam, it was ${\hat{S}}_{z}+{\hat{L}}_{z}=(n+1)W$. During focusing, the total spin of the beam (28) decreased in the focal plane, while the total OAM increased in it:

(35) $$\begin{array}{l} {\hat{S}}_{z}=\left(W_{0}-W_{2}\right)=W-2\left(W_{2}+W_{1}\right),\;\;\\ {\hat{L}}_{z}=nW+2\left(W_{2}+W_{1}\right). \end{array}$$

This effect is called SOC [4]. Therefore, if the initial field (1) has no spin (no SAM), then there is no SOC in the focus and the total spin is zero (10): ${\hat{S}}_{z}=0$. However, the spin Hall effect (9) can be formed at the focus. The OAM for the field (1) is also preserved (14) and is equal to ${\hat{L}}_{z}=nW$, and there is an orbital Hall effect ((12) and (13)) at the focus. If there is an SAM (29) in the initial field (28), then due to the SOC, it is not kept in focus, but decreases, according to (35), partially converting into OAM. The beam (28) also has spin and orbital Hall effects [20]. However, both of these effects are radial, i.e., the sign of the SAM and the OAM is different at different radii from the optical axis.

## 9. Conclusions

In this study, the following results were obtained. It was shown that during tight focusing of an optical vortex with an arbitrary radially symmetric amplitude function and with linear polarization, the distribution of the SAM axial component (9) in the focal plane depends on the azimuthal angle *φ* according to cos(2*φ*), and therefore, for a TC *n* ≠ 0, the spin Hall effect takes place at the focus. This effect leads to the formation of two regions on the vertical and horizontal axes in which the polarization vector rotates in different directions (clockwise and counterclockwise) and SAM has different signs. Similarly, it was derived that the OAM axial component (12) depends on the azimuth angle *φ* according to cos(2*φ*) at the focus. However, we cannot call these four regions with different signs of the OAM longitudinal component a manifestation of the orbital Hall effect since we do not know how the transverse energy flow behaves in these regions (changes the direction of rotation or not). It was also demonstrated that the transverse energy flux rotates in the plane of focus in opposite directions at different radii from the optical axis (5). Such a distribution of the transverse energy flux at the focus can be called the radial–orbital Hall effect since the energy flux will rotate dielectric microparticles trapped at different radii at the focus clockwise or counterclockwise (the angular tractor [31]).
